# Viewpoint: Assessing the value of mental health treatments in Europe

**DOI:** 10.1192/j.eurpsy.2023.2419

**Published:** 2023-08-09

**Authors:** Judit Simon, Patrice Boyer, Jose M. Caldas-de-Almeida, Martin Knapp, Paul McCrone, Philip Gorwood, Wolfgang Oertel, Celso Arango, Janet Treasure, Allan H. Young, Frederic Destrebecq, Vinciane Quoidbach

**Affiliations:** 1 Department of Health Economics, Center for Public Health, Medical University of Vienna, Vienna, Austria; 2 Department of Psychiatry, University of Oxford, Oxford, UK; 3 European Brain Council and European Brain Foundation, Brussels, Belgium; 4 Lisbon Institute of Global Mental Health, Comprehensive Health Research Centre, Nova Medical School, Nova University of Lisbon, Lisbon, Portugal; 5 Care Policy and Evaluation Centre, London School of Economics and Political Science, London, UK; 6 Greenwich University, London, UK; 7 GHU Paris Psychiatrie et Neurosciences (CMME), INSERM UMR1266, Université Paris Cité, Paris, France; 8 Philipps-Universität Marburg, Marburg, Germany; 9 Department of Child and Adolescent Psychiatry, Institute of Psychiatry and Mental Health, Hospital General Universitario Gregorio Marañón, IiSGM, School of Medicine, Universidad Complutense, CIBERSAM, Madrid, Spain; 10 Department of Psychological Medicine, Institute of Psychiatry, Psychology and Neuroscience, King’s College London, London, UK; 11 South London and Maudsley NHS Foundation Trust, London, UK; 12 Bethlem Royal Hospital, Beckenham, UK

**Keywords:** mental health, cost-effective early detection and intervention, prevention, value-based health care

## Abstract

One in eight individuals worldwide lives with a mental health disorder. For many European countries, the prevalence is even higher, with one in four people reporting mental health problems [1]. Three-quarters of all mental health disorders develop before age 25, with many presenting initially in undiagnosed forms already in the mid-teens and eventually manifesting as severe disorders and lasting into old age [2]. There is also growing evidence that mental health disorder symptoms cross diagnoses and people frequently have more than one mental health disorder [3].

One in eight individuals worldwide lives with a mental health disorder. For many European countries, the prevalence is even higher, with one in four people reporting mental health problems [1]. Three-quarters of all mental health disorders develop before age 25, with many presenting initially in undiagnosed forms already in the mid-teens and eventually manifesting as severe disorders and lasting into old age [2]. There is also growing evidence that mental health disorder symptoms cross diagnoses and people frequently have more than one mental health disorder [3].

Mental health problems are not only prevalent, enduring in nature and complex, but also closely interconnected with many aspects of life, and therefore disabling. They increase the risk for school drop-out, are a major cause of work absenteeism, presenteeism, and incapacity to work, are closely related to poverty, negatively impact family lives, increase social isolation, and decrease life expectancy by 15 to 20 years, mostly due to somatic disorders [4]. According to the latest Global Burden of Disease estimates, depression, anxiety, substance abuse disorders, and schizophrenia are among the 25 leading causes of overall disease burden between ages 10 and 49 years in the world [5]. The mental health impact of the coronavirus disease-2019 (COVID-19) pandemic and related measures further increased the occurrence of depression and anxiety by about one-quarter [6]. Additionally, longitudinal studies indicate multiple sustained adverse effects on mental health – especially for those with existing mental health problems, young people, older people and women – requiring longer-term coordinated responses within and beyond health care [7].

Already before the COVID-19 pandemic, the societal cost of mental health disorders exceeded 4% of GDP (over EUR 600 billion) across the 28 European Union countries. Direct health care costs (EUR 190 billion) were estimated overall to be lower than the economic impacts of lower employment and productivity of people with mental health issues (EUR 260 billion), similar in size to spending on social security programs (EUR 170 billion) [8]). While substantially diverse across healthcare segments and ill-health conditions, excess healthcare costs associated with the increased risks of physical comorbidities linked to mental health disorders have been found to be between 37 and 110%, calling for more tailored policy considerations toward integrated care options [4]. Furthermore, the economic value of life years lost due to morbidity and mortality linked to mental health disorders exceeds their cost impact: recently, this was valued as between 6 and 7.7% of GDP in Europe, suggesting potentially large returns on investment from improved prevention, detection and treatment of mental health problems [9].

Although effective and cost-effective early detection and interventions exist, there are numerous unmet needs along the mental health care pathways. Navigating the mental health services system is often complex for patients and families due to fragmented or disrupted services (in-patient and outpatient care) and discontinuity of care (transition between child and adult mental health services, for example) [10]. Policies and programs to prevent and mitigate the negative impacts on schooling, employment, families and risky behaviors are also paramount. Nevertheless, spill-over effects to other economic sectors (work, education, legal system, and informal care) are usually neglected in economic burden and value assessments. Current value estimates usually also remain limited in terms of comprehensive outcome assessment for patients and families [11], and comprehensive and comparable societal cost impact assessment [12] due to the lack of relevant data.

## Value of treatment

Value-based healthcare is gaining traction in Europe as the desired solution or path forward in improving health systems. Achieving high value for patients must become the overarching goal of health care delivery, with value defined as the health outcomes achieved per money spent [13]. The approach towards more comprehensive mental health care models critically intertwines wider patient and societal outcomes with efficient spending of resources. Reinforcing this should lead to both better care for patients and a more sustainable framework for payers.

In 2019, the European Brain Council (EBC) initiated a second research project on the Value of Treatment (VOT2) focused on early detection and continuity of care for persons living with selected mental disorders. The research project – which combines care pathways analysis and economic analyses – included three case studies related to anorexia nervosa (AN), autism spectrum disorder (ASD), and major depressive disorder (MDD). It aimed to identify treatment gaps, assess the potential outcomes and costs of optimized care, and provide policy recommendations. Case studies were analyzed in collaboration with EBC’s scientific societies and patient organizations in line with the research framework.

## Case studies

In light of the complexity of mental health care pathways and their national variations, these case studies focused on specific aspects of early intervention and continuity of care provision, including the transition of care for AN, early intervention including associated epilepsy in ASD, and the value of an optimal stepped care model for MDD. Mapping surveys in multiple European countries demonstrated major treatment gaps. Those surveys were followed by an assessment of the potential health and healthcare cost impacts of closing these gaps in optimized care pathway models (through the stages of screening, diagnosis, treatment, and follow-up) and resulted in care improvement recommendations. All three case studies found:

- lack or delayed detection in more than 50% of cases,
- long waiting times and duration of untreated illnesses including over one-year delays in access to screening and diagnosis (ASD), or specialist treatment (AN),
- fragmentation and lack of collaboration between services offering different treatment options,
- disruption of the continuity of care between various service levels and along the life course,
- large within- and between-country service and practice variations,
- high out-of-pocket expenses for patients and families,
- lack of organized support for families caring for children impacted by the disorders, and
- very limited systematic and comparable data on epidemiology, guideline adherence, patient and family outcomes, and costs, limiting robust socio-economic assessments.

In the case of AN, reducing the three main treatment gaps by 50% in terms of improved waiting times, specialist service access, and transition support could reduce the disease burden substantially. Early diagnosis and medication treatment of autistic children with epilepsy was shown to be cost-effective, and country-specific strategies would make the economic case even stronger. Reducing treatment gaps in the detection and treatment of MDD is likely to achieve better outcomes at increased short-term costs. However, investigated optimization strategies for MDD represent overall good value at the currently applied cost-effectiveness thresholds across all modeled countries if implementation costs remain below certain limits.

## Opportunities for optimized and harmonized actions

The VOT2 case studies found that, although established clinical guidelines are available for AN, ASD, and MDD, there are major geographical care variations in Europe, leading to reduced effectiveness and efficiency and substantial negative health impacts. Due to the lack of relevant surveillance and monitoring tools, the magnitude of these care variations remains unknown. Furthermore, outcome and cost estimates remain limited due to the underdeveloped evidence base. Nevertheless, the case studies confirm the added value of early detection and intervention and the implementation of a continuous and comprehensive healthcare approach as opposed to fragmentation in separate medical (and non-medical) “silos.” Analyzing multiple diseases jointly also made the resulting conclusions more impactful for public health and reinforced the call for pan-European actions.

**Figure 1.**
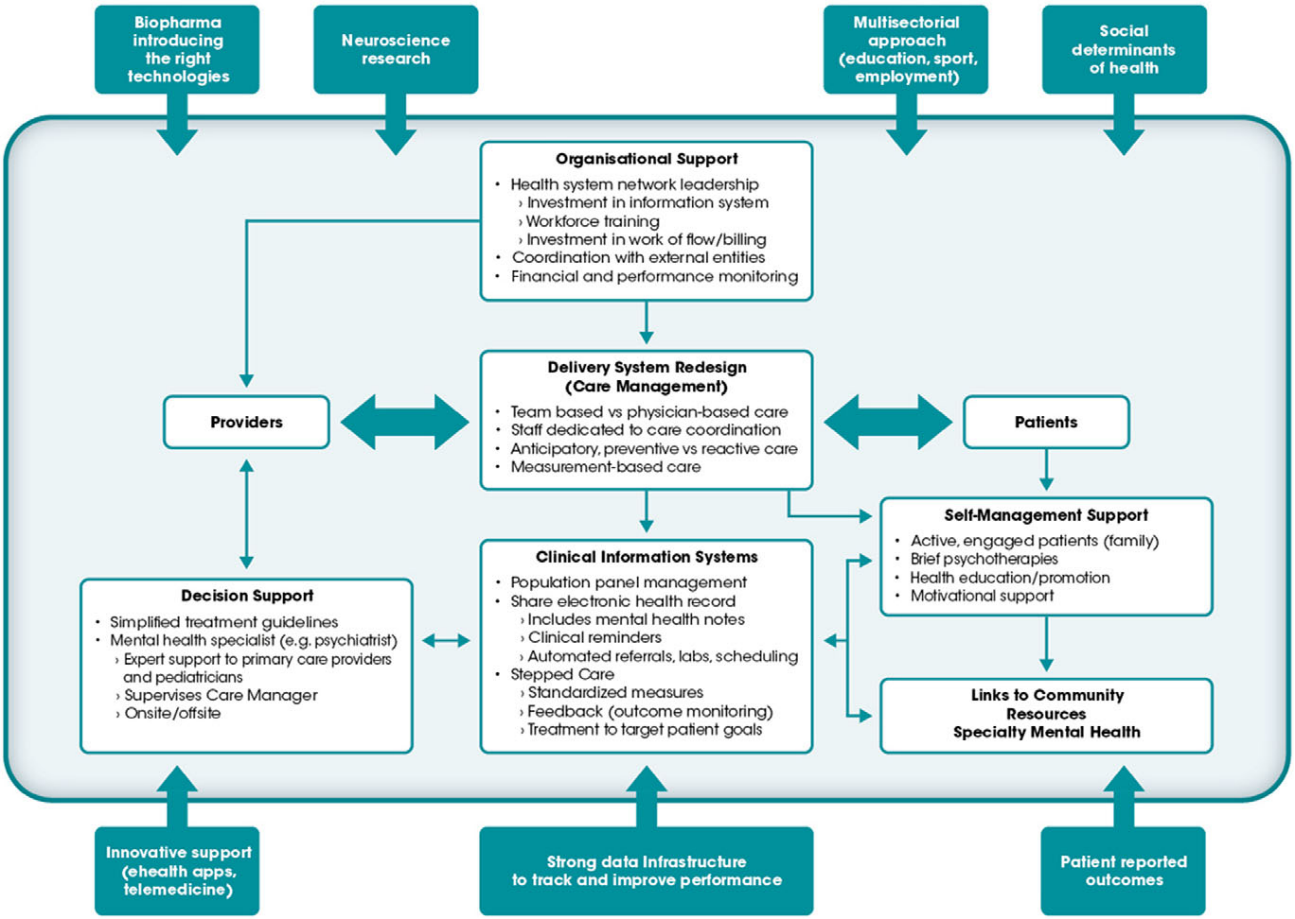
An enabling ecosystem for bridging gaps through increased early intervention and continuity of care. *Source*: The figure is a replication from the Case Study Results & Call to Action: Mental Disorders (March 2022) with permission from the European Brain Council (EBC).

On the other hand, harmonization towards one “gold standard” European care pathway that includes early detection and intervention for mental disorders is unlikely to be optimal or feasible. When considering that the richest and the poorest EU-27 countries vary in their relative GDP per person by almost five-fold, there are non-negligible differences in terms of what services and interventions may be seen as the best value and affordable in the given healthcare system emerge. Future efforts towards optimal mental health care pathways will require both explicit value and affordability considerations and the alignment of relevant public reimbursement plans in order to reduce the already immense economic burden on patients and families. Some elements of an enabling ecosystem, however, remain universal as shown in Figure 1.

Defined EU policy areas around healthcare systems and related policy/regulatory initiatives include:

- Value-based digital care including technology and digital treatments (e.g., EU Digital Health Strategy)
- New treatment paradigms and access (e.g., EU Pharma Strategy, Joint Clinical Assessment for Health Technology Assessment)
- Patient engagement through personalized treatment and support, and the use of Patient-Reported Outcomes (PROs) (e.g., ICHOM)
- Patient participation in key clinical decisions and policy discussions
- Collaboration in monitoring and research via data sharing and infrastructure (e.g., European Research Networks, European Health Data Space)

The above-identified research and public health policy gaps and opportunities at the EU level need to be addressed in combination with harmonized, national-level actions. These should include country-specific guideline adaptations, integrated service optimization, the development of adaptive payment schemes, improved routine care, outcome and cost data collection and linkage through national registries, (bio)data banks and cost databases, and greater investment in mental health promotion and mental ill-health prevention beyond the healthcare system, especially during early childhood and adolescence. Building a strong foundation for mental health and investing in optimal interventions tailored to the young generation advances socio-economic development. Hopefully, this will be addressed by the European Commission in the development of a comprehensive strategy for mental health in the EU to be released during the second quarter of 2023.
